# What does genomic medicine mean for diverse populations?

**DOI:** 10.1002/mgg3.63

**Published:** 2014-01-10

**Authors:** Adebowale Adeyemo, Charles Rotimi

**Affiliations:** Center for Research on Genomics and Global Health, National Human Genome Research Institute, National Institutes of HealthBethesda, Maryland, 20892

The completion of the effort to sequence the human genome opened up a new era in human genomics. The new tools and technologies developed, as well as new models for collaboration, data sharing, and data release provided a good foundation for the international projects that attempted to characterize genomic variation in diverse populations. The first of these global efforts, the International HapMap Project (http://www.hapmap.org), generated a catalog of common genetic variation in eleven global populations reflecting different ancestries. The 1000 Genomes Project (http://www.1000genomes.org) greatly expanded the scope of known human variation by sequencing over 2500 individuals from 26 global populations. The data from these projects, as well as from other projects of more limited scope, yielded an unprecedented knowledge of the scope of human genetic variation, provided data on selective forces on the human genome and led to new research designs and technologies (e.g., genome-wide association studies [GWAS], whole exome capture/sequencing and whole genome sequencing) for finding disease loci. The ever-mounting volume of new discoveries of disease-associated loci is providing unprecedented insights into biology and suggesting paths to clinical translation, leading to the growing acknowledgment of the dawn of the era of genomic medicine (Muenke 2013).

Genomic medicine, or the use of information from genomes and their derivatives (RNA, proteins, and metabolites) to guide medical decision-making (Ginsburg and Willard 2009), is now considered a key component of personalized medicine. The potential of genomic tools to inform clinical medicine is defined by the extent to which it can be applied to predicting risk of disease, natural history, treatment response, and risk of adverse reactions to pharmaceutical agents (among others). Thus, a fundamental requirement for the successful implementation of genomic science in the practice of medicine is the availability of empirical evidence generated from research. It is gratifying that each new development in genomics has been followed by studies that apply the new tools and technologies to the study of disease and treatment response. For example, the International HapMap Project was swiftly followed by GWAS, which have now been applied to scores of diseases, resulting in over 1700 publications as of December 2013 (NHGRI GWAS catalog – http://www.genome.gov/26525384), with implications for disease risk, pathogenesis, and treatment response. However, the vast majority of these applied studies have been performed only in European ancestry populations (Hindorff et al. 2009; Need and Goldstein 2009) and most of the population diversity present globally are inadequately represented. For example, the NHGRI GWAS catalog shows that non-European populations comprise just a small fraction of GWAS studies (Fig. 1).

**Figure 1:**
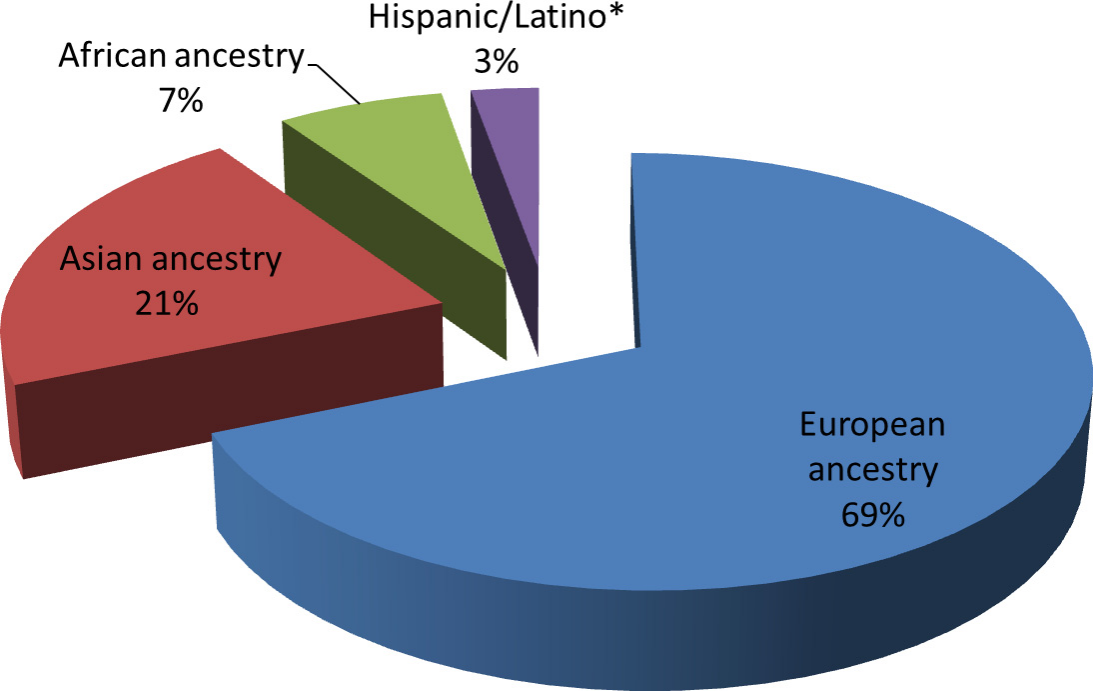
GWAS publications by ancestry of discovery sample. Source of data: NHGRI GWAS catalog – http://www.genome.gov/26525384 – accessed 11 December 2013 (*n* = 1774 GWAS publications). Note that early studies did not consistently label ancestry. Also, some discovery studies used multiple ancestries. Therefore, the “European ancestry” category is an underestimate. *“Hispanic/Latino” is not an ancestry, but an ethnic group. It is used here as a population label that includes “Hispanic,” “Latino,” “Mexican American,” and similar labels.

Given that population groups have similarities but also exhibit differences, this implies that the evidence base for genomic medicine is missing for most non-European ancestry populations. To what extent should this be a concern? Several studies show that GWAS-associated loci exhibit marked differences in allele frequencies between global populations (Adeyemo and Rotimi 2010) and in effect size (Ntzani et al. 2012), with the latter study showing point estimates of risks were opposite in direction or differed more than twofold in 57%, 79%, and 89% of the European versus Asian, European versus African, and Asian versus African comparisons, respectively. A recent study (Carlson et al. 2013) further emphasized these differences, showing that at least a quarter of tag SNPs identified in European American GWAS had significantly different effect sizes in at least one non-European ancestry population, with African Americans showing differential effects most frequently. This implies that most genetic-risk models derived from GWAS findings in European Americans may generate spurious findings when applied to other populations. Specific diseases also tend to show the same pattern. For example, the KCNQ1 locus for type 2 diabetes (T2D) was first discovered in East Asians (Unoki et al. 2008; Yasuda et al. 2008), having been missed in previous studies of European ancestry populations due to the low allele frequency in the latter (5% vs. 40%). On the other hand, the TCF7L2 T2D locus shows a reciprocal effect (3% in East Asians vs. 30% in Europeans) (McCarthy 2008). Indeed, it was subsequently shown that T2D risk loci, when compared to other diseases, demonstrate extreme differentiation among populations (Chen et al. 2012).

In contrast to diseases that tend to occur only in specific population groups and are rare to be absent in others (e.g., sickle cell disease, cystic fibrosis), certain diseases and conditions may show less marked disparity in incidence, but the underlying genetic-risk factors could be quite instructive. A good example is end-stage renal disease (ESRD), which is four times more prevalent in African Americans compared to European Americans. Admixture mapping led to the identification of a chromosome 22 locus, which on further refinement led to the *APOL1* as a major genetic-risk factor for ESRD (Genovese et al. 2010). The frequency of the risk variant (rs73885319) shows tremendous variation worldwide: 40% among the Yoruba from Nigeria (West Africans), 20% in African Americans, and 0% in Europeans and East Asians. The disease risk variant is believed to have risen to high frequency in Africa because it protects against the lethal form of African sleeping sickness. This example illustrates the potential utility of studying non-European ancestry populations, especially as the initial discovery was through admixture mapping, which would be inapplicable in a homogenous or nonadmixed population.

Personalized medicine involves being able to optimize drug selection, dose, and treatment duration, while averting adverse drug reactions for the individual patient. Genetic variants can serve as useful biomarkers for the absorption, distribution, metabolism, and excretion (ADME) of specific drugs. However, our understanding of the distribution of human pharmacogenomic variation remains limited and there persists poor representation of ethnically diverse samples from various parts of the world in such studies. Global studies of ADME genes (Li et al. 2011; Ramos et al. 2013) demonstrate wide differences among populations in these variants that have such a high clinical importance. Specific examples are particularly instructive. Warfarin, the most commonly used anticoagulant worldwide, is characterized by a narrow therapeutic index and wide inter and intraindividual variation in the dose required for the target therapeutic response. Genetic variation in *VKORC1* and *CYP2C9* explain ∼30–60% of the variation in therapeutic warfarin dose in European and Asian populations, but explain less variability for individuals of African descent. This difference is largely driven by allele frequency differences (Limdi et al. 2010; Suarez-Kurtz and Botton 2013). A recent GWAS in African Americans (Perera et al. 2013) identified a novel CYP2C single-nucleotide polymorphism that has a clinically relevant effect on warfarin dose in African Americans, independent of the previously described CYP2C9*2 and CYP2C9*3 variants. This implies that incorporation of this variant into pharmacogenetic dosing algorithms could improve warfarin dose prediction for this specific population. Other examples exist for: childhood acute lymphoblastic leukemia, which shows marked ethnic differences in survival and for which a recent study found that Native American ancestry was associated with risk of relapse and modifications to treatment mitigated this ancestry-related risk of relapse (Yang et al. 2011); and hepatitis C virus treatment response and spontaneous clearance, for which an IL28B polymorphism explains at least half of the difference in response rates observed in Caucasians and African–Americans who received the same treatment with comparable adherence (Ge et al. 2009).

Therefore, the available evidence suggests that: (1) populations often show considerable differences at clinically relevant loci, including disease risk loci and loci that predict drug response; (2) most studies that produce data that can be useful for genomic medicine have been carried out largely in European ancestry populations. Thus, the evidence base for genomic medicine in such diverse populations is lacking and lags far behind that of European ancestry populations. For such populations, the advantages of genomic medicine may remain largely theoretical and out of reach until the necessary research is done to improve the evidence base applicable to them.

The need for more genomic research in diverse populations is obvious. The last few years have seen multiple initiatives to address this issue. In the United States, these have included building genomics studies on existing epidemiological cohort studies, funding specific research grants to study US minority populations and studies specifically targeted at multiethnic populations. An example of the latter is the Population Architecture Using Genomics and Epidemiology (PAGE) study, which is based on a consortium of multiancestry, population-based studies formed with the objective of refining the genetic architecture of common traits emerging from GWAS (https://www.pagestudy.org/). Regional and/or country genomic initiatives now exist in Asia, Central and South America, and Africa. Africa, the continent with the greatest human genetic diversity but the least studied, is currently the site of a new genomics initiative: the Human Heredity and Health in Africa Initiative (H3Africa, http://h3africa.org/) jointly funded by the US National Institutes of Health and the UK Wellcome Trust. The initiative is building genomics capacity and infrastructure on the continent, while funding multiple high-quality research projects into T2D, cardiometabolic disease, rheumatic heart disease, chronic kidney disease, neurological diseases, microbiomes, several infectious diseases, and pharmacogenomics (among others) – http://h3africa.org/projects. The findings have the potential to inform genomic medicine not just in Africa but globally.

A trend which could accelerate the relevance of genomic medicine to diverse populations is the increasing number of genomic studies and initiatives based on electronic medical records (EMR). Such studies and initiatives have the potential to explore multiple facets of genomic medicine, including development and refinement of the evidence base, defining and testing clinical validity and actionability, integration of genomic findings into routine clinical practice, figuring out how best to achieve physician uptake and studying return of results to patients. As such studies are based on patient populations, they have the potential to include diverse populations to the extent to which they have access to the health care system in which the study is being conducted. A good example of such an initiative is the eMERGE (Electronic Medical Records and Genomics) Network (http://emerge.mc.vanderbilt.edu/), a consortium funded by the National Human Genome Research Institute to develop, disseminate, and apply approaches to genomic research. In the first phase (2007–2011), the consortium investigated the use of EMRs and biorepositories in genomic research including conducting GWAS for 13 phenotypes on ∼19,000 genotyped participants resulting in the identification of 12 novel loci and replication of 16 loci. In the second phase (2011–2014), the network is studying the incorporation of genomic variants into EMRs for use in clinical care and will conduct GWAs for 24 additional phenotypes on a total of ∼87,000 genotyped participants. Such studies with diverse groups of genotyped participants linked to EMRs could be highly useful in generating new data, while testing the feasibility of using genomic data in clinical care. In particular, issues that may differ between ethnic groups such as notions of privacy, community engagement, genetic counseling, and return of results may be better addressed within such networks. These studies will be facilitated by the increasing drive to make EMRs universal and to provide improved access to health care to as many people as possible.

In conclusion, considerable progress has been made in the push to implement genomic medicine as a crucial part of personalized medicine. However, non-European ancestry populations lag behind European ancestry populations because of limited participation in genomic research both as research subjects and as research investigators. These limitations have resulted in a poorer evidence base for genomic medicine in diverse populations. While more research is needed, several ongoing initiatives could provide the data to improve the evidence base and make genome medicine useful to diverse populations.

## References

[b1] Adeyemo A, Rotimi C (2010). Genetic variants associated with complex human diseases show wide variation across multiple populations. Public Health Genomics.

[b2] Carlson CS, Matise TC, North KE, Haiman CA, Fesinmeyer MD, Buyske S (2013). Generalization and dilution of association results from European GWAS in populations of non-European ancestry: the PAGE study. PLoS Biol.

[b3] Chen R, Corona E, Sikora M, Dudley JT, Morgan AA, Moreno-Estrada A (2012). Type 2 diabetes risk alleles demonstrate extreme directional differentiation among human populations, compared to other diseases. PLoS Genet.

[b4] Ge D, Fellay J, Thompson AJ, Simon JS, Shianna KV, Urban TJ (2009). Genetic variation in IL28B predicts hepatitis C treatment-induced viral clearance. Nature.

[b5] Genovese G, Friedman DJ, Ross MD, Lecordier L, Uzureau P, Freedman BI (2010). Association of trypanolytic ApoL1 variants with kidney disease in African Americans. Science.

[b6] Ginsburg GS, Willard HF (2009). Genomic and personalized medicine: foundations and applications. Transl. Res.

[b7] Hindorff LA, Sethupathy P, Junkins HA, Ramos EM, Mehta JP, Collins FS (2009). Potential etiologic and functional implications of genome-wide association loci for human diseases and traits. Proc. Natl. Acad. Sci. USA.

[b8] Li J, Zhang L, Zhou H, Stoneking M, Tang K (2011). Global patterns of genetic diversity and signals of natural selection for human ADME genes. Hum. Mol. Genet.

[b9] Limdi NA, Wadelius M, Cavallari L, Eriksson N, Crawford DC, Lee MT (2010). Warfarin pharmacogenetics: a single VKORC1 polymorphism is predictive of dose across 3 racial groups. Blood.

[b10] McCarthy MI (2008). Casting a wider net for diabetes susceptibility genes. Nat. Genet.

[b11] Muenke M (2013). Individualized genomics and the future of translational medicine. Mol. Genet. Genomic Med.

[b12] Need AC, Goldstein DB (2009). Next generation disparities in human genomics: concerns and remedies. Trends Genet.

[b13] Ntzani EE, Liberopoulos G, Manolio TA, Ioannidis JP (2012). Consistency of genome-wide associations across major ancestral groups. Hum. Genet.

[b14] Perera MA, Cavallari LH, Limdi NA, Gamazon ER, Konkashbaev A, Daneshjou R (2013). Genetic variants associated with warfarin dose in African-American individuals: a genome-wide association study. Lancet.

[b15] Ramos E, Doumatey A, Elkahloun AG, Shriner D, Huang H, Chen G (2013). Pharmacogenomics, ancestry and clinical decision making for global populations. Pharmacogenomics J.

[b16] Suarez-Kurtz G, Botton MR (2013). Pharmacogenomics of warfarin in populations of African descent. Br. J. Clin. Pharmacol.

[b17] Unoki H, Takahashi A, Kawaguchi T, Hara K, Horikoshi M, Andersen G (2008). SNPs in KCNQ1 are associated with susceptibility to type 2 diabetes in East Asian and European populations. Nat. Genet.

[b18] Yang JJ, Cheng C, Devidas M, Cao X, Fan Y, Campana D (2011). Ancestry and pharmacogenomics of relapse in acute lymphoblastic leukemia. Nat. Genet.

[b19] Yasuda K, Miyake K, Horikawa Y, Hara K, Osawa H, Furuta H (2008). Variants in KCNQ1 are associated with susceptibility to type 2 diabetes mellitus. Nat. Genet.

